# Neurogenic inflammation after traumatic brain injury and its potentiation of classical inflammation

**DOI:** 10.1186/s12974-016-0738-9

**Published:** 2016-10-11

**Authors:** Frances Corrigan, Kimberley A. Mander, Anna V. Leonard, Robert Vink

**Affiliations:** 1 Adelaide Centre for Neuroscience Research, School of Medicine, The University of Adelaide, Adelaide, South Australia Australia; 2 Sansom Institute for Health Research, The University of South Australia, Adelaide, South Australia Australia

**Keywords:** Caveolae, Neuroinflammation, Neurokinin 1 receptor, Substance P, Traumatic brain injury

## Abstract

**Background:**

The neuroinflammatory response following traumatic brain injury (TBI) is known to be a key secondary injury factor that can drive ongoing neuronal injury. Despite this, treatments that have targeted aspects of the inflammatory pathway have not shown significant efficacy in clinical trials.

**Main body:**

We suggest that this may be because classical inflammation only represents part of the story, with activation of neurogenic inflammation potentially one of the key initiating inflammatory events following TBI. Indeed, evidence suggests that the transient receptor potential cation channels (TRP channels), TRPV1 and TRPA1, are polymodal receptors that are activated by a variety of stimuli associated with TBI, including mechanical shear stress, leading to the release of neuropeptides such as substance P (SP). SP augments many aspects of the classical inflammatory response via activation of microglia and astrocytes, degranulation of mast cells, and promoting leukocyte migration. Furthermore, SP may initiate the earliest changes seen in blood-brain barrier (BBB) permeability, namely the increased transcellular transport of plasma proteins via activation of caveolae. This is in line with reports that alterations in transcellular transport are seen first following TBI, prior to decreases in expression of tight-junction proteins such as claudin-5 and occludin. Indeed, the receptor for SP, the tachykinin NK1 receptor, is found in caveolae and its activation following TBI may allow influx of albumin and other plasma proteins which directly augment the inflammatory response by activating astrocytes and microglia.

**Conclusions:**

As such, the neurogenic inflammatory response can exacerbate classical inflammation via a positive feedback loop, with classical inflammatory mediators such as bradykinin and prostaglandins then further stimulating TRP receptors. Accordingly, complete inhibition of neuroinflammation following TBI may require the inhibition of both classical and neurogenic inflammatory pathways.

## Background

The role of inflammation in perpetuating the secondary injury response following traumatic brain injury (TBI) has received a significant amount of attention over the last two decades and is clearly an important factor in exacerbating neuronal injury. However, while many pre-clinical studies have shown that therapeutics targeting the immune response are effective in improving outcome when administered in the immediate aftermath of the injury [1–3], reports from clinical trials have been less promising. Agents with known anti-inflammatory properties such as corticosterone, progesterone, and erythropoietin (EPO) have all shown no benefit to date [4–6]. Specifically, the CRASH trial, a prospective, randomized, placebo-controlled multicenter trial of the corticosteroid, methylprednisolone, in TBI, reported an increased mortality following TBI [4]. The ProTECT III trial utilizing progesterone was halted due to failure to demonstrate improved outcome by the Glasgow Outcome Scale-Extended Score at 6 months post-injury [6], with similar findings in another clinical trial utilizing progesterone, the SyNAPse study [5]. In addition, promising anti-inflammatory agents identified in pre-clinical studies often have narrow therapeutic windows. For example, interleukin-1 antagonists appear most efficacious when first administered within hours following injury [1, 2], with minocycline also typically delivered within the first hour post-injury [7–9]. This may be, in part, due to the duality of the immune response following TBI, with some aspects of the inflammatory response necessary to promote repair [10]. In addition, this may also reflect that classical inflammation may be only be half the story, with neurogenic inflammation recently reported as playing a key initiating role [11–13], augmenting many aspects of the classical inflammatory response [14, 15]. This review will outline the interrelationship between classical and neurogenic inflammation, promoting a better understanding of the entire neuroinflammatory cascade and potentially facilitating the development of targeted anti-inflammatory regimes that can improve outcome following TBI.

## Traumatic brain injury

TBI results from the head impacting with an object or from acceleration/deceleration forces that produce vigorous movement of the brain within the skull or varying combinations of these mechanical forces [16]. The resultant injury is caused by two mechanisms, either primary or secondary, although there is some degree of overlap [17, 18]. Primary injury is the result of mechanical forces (rotation, acceleration/deceleration, and direct force applied to the head) acting at the moment of the injury that damage the blood vessels, axons, nerve cells, and glia of the brain in a focal, multifocal, or diffuse pattern of involvement. The type and severity of the resulting injury depends upon the nature of the initiating force, as well as the site, direction, and magnitude of the force [19]. Contact forces generated when the head strikes or is struck by an object generally produce focal injuries, such as skull fractures, extradural hemorrhages, and contusions. In contrast, acceleration/deceleration forces that result from violent unrestrained head movement, such as in a motor vehicle accident, are associated with diffuse axonal injury (DAI) [20]. In contrast, secondary injury is a gradual process that occurs over minutes to days as the result of cellular, neurochemical, and metabolic alterations initiated by the primary insult [21]. Injury factors that contribute to this phenomenon include metabolic changes, edema formation, calcium influx, increased oxidative stress, excitotoxicity, inflammation, and ultimately, cell death via necrosis or apoptosis [22]. In particular, inflammation is thought to contribute to much of the secondary cell injury, directly injuring cells, and facilitating other injury factors such as oxidative stress [23] and edema formation [24, 25].

## Classical inflammatory response following TBI

A robust inflammatory response develops acutely post-TBI and is characterized by the activation of resident cells, migration and recruitment of peripheral leukocytes, and the release of inflammatory mediators [26]. Cellular damage associated with the mechanical impact causes the release of a number of endogenous factors such as RNA, DNA, heat shock proteins, and HMGB1 (high mobility group box 1) which act as damage-associated molecular patterns (DAMPs) [27]. These bind to toll-like receptors (TLRs) activating the nuclear-factor-κB (NFκB) and MAPK pathways leading to the release of a variety of pro-inflammatory factors including cytokines (IL-1β, IL-6), chemokines, and immune receptors [28]. Members of the TLR family are expressed by a number of resident cells within the central nervous system (CNS), including astrocytes, microglia, and the cerebrovascular endothelium [29–31]. Apart from DAMPs, the classical inflammatory response is also initiated by the presence of extravasated blood products, complement fragments, and reactive oxygen and nitrogen species [27, 32, 33].

This inflammatory response is signaled by a rapid rise in the levels of cytokines and chemokines following TBI, with release from microglia, astrocytes, cerebrovascular endothelial cells, peripheral immune cells, and even neurons [32, 34, 35]. Following a moderate diffuse TBI in mice, for example, levels of IL-1β, tumor necrosis factor alpha (TNFα) and IL-6 within the cortex peak at 3–9 h post-injury, before gradually subsiding [36]. Similarly, within clinical studies, increased levels of IL-6, TNFα, IL-10, C-C motif chemokine ligand 2 (CCL2), and IL-8 peak within the first 2 days following moderate-severe TBI and then return to normal over a period of several weeks [37–39]. This spike in cytokine release has been correlated with astrogliosis, microglial activation, and axonal dysfunction, providing evidence of the association between the activated immune response and brain pathology [40].

Immune cells are recruited to the area of injury by the release of chemokines from the damaged neuronal tissue [41, 42]. This cellular response to injury appears to differ slightly depending on whether the initiating insult is primarily focal or diffuse in nature. A focal injury is characterized by the early infiltration of neutrophils (peaking within a few days), followed by the migration of microglia, astrocytes, macrophages, and lymphocytes to the injured site [43]. Flow cytometric analysis indicates that there is a 10- to 20-fold increase in numbers of microglia compared to peripheral macrophages, suggesting that this is predominantly a central rather than peripheral response [44]. In diffuse injury, little to no neutrophil infiltration is seen, with the early cellular response consisting of microglial accumulation and astrocytosis most prominent in the white matter tracts [45]. This response amplifies over-time with Hellewell et al. indicating that the highest numbers of microglia are present at 14 days post-injury, the latest time-point investigated in their study [45]. Even mild TBI is associated with the induction of an inflammatory response, with diffuse mTBI in pigs showing enhanced microglial activation associated with thalamic axonal injury at 6 h post-injury [46].

The function of this inflammatory response can be both detrimental and potentially beneficial. Both microglia and astrocytes can serve a neuroprotective role immediately following injury by clearing damaged cell debris by phagocytosis, releasing anti-inflammatory cytokines and neurotrophic factors, [33, 47, 48]. Indeed, numerous studies have shown that at least some inflammation is necessary following an insult to the CNS to assist in clearing damage and preparing for remodeling [10, 26]. For example, ablation of proliferating reactive astrocytes following a moderate controlled cortical impact injury significantly exacerbated cortical neuronal loss and inflammation [49]. A potential protective role for astrocytes following injury includes removal of glutamate to reduce the effects of excitotoxicity [50], with the glial scar also thought to act as a physical barrier to prevent spread of toxic molecules. [51] However, this glial scar can also have a later inhibitory effect on axonal regrowth and regeneration [52, 53]. Furthermore, even pro-inflammatory cytokines have an important role to play with Scherbel et al. showing that although knockout of TNFα was beneficial in the acute phase following injury, it had long-term deleterious consequences as demonstrated by TNFα−/− mice showing worsening of motor outcome at 1 month following a focal TBI, which was associated with enhanced cortical tissue loss [54]. This may relate to purported associated neuroprotective functions of TNFα, including the ability to reduce oxidative stress [55] and promote neurotrophic factor synthesis [56, 57], indicating that the presence of at least some TNFα is needed following injury.

In addition, activated microglia demonstrate phenotypic subpopulations, characterized by a specific molecular signature of gene expression: M1 microglia promote a classic pro-inflammatory state releasing pro-inflammatory cytokines and oxidative metabolites, while M2 microglia are important for tissue remodeling and suppress the inflammatory response [58–60]. Reports from TBI studies suggest that there is an early peak in M2-like activated microglia in the week following injury [61, 62], but this then shifts to a maladaptive M1-like activation at later time points. The importance of M2 microglia after TBI is demonstrated by a study by Kumar et al., where aged mice with an impaired M2 response had increased lesion size following a focal injury [63]. Indeed, M1 activation can exacerbate neuronal injury by triggering downstream pathways that culminate in oxidative damage, activation of apoptotic cell death, and increases in permeability of the blood-brain barrier (BBB) through modifications in its tight junctions (Tight junctions) and matrix metalloproteinase (MMP) activation [64, 65]. Furthermore, prolonged M1-like activation hampers repair and can allow tissue damage to persist for years after the initial injury; in a subset of TBI patients, there is incomplete resolution of the acute neuroinflammatory response [66]. A vicious cycle is initiated following the original insult, where the release of pro-inflammatory factors by resident glial cells promotes further glial activation, leading to a progressive chronic cycle of neuroinflammation [67], which can have neurotoxic effects on neurons through mechanisms such as oxidative stress, apoptosis, and excitotoxicity [68].

## Neurogenic inflammation

Activation of sensory unmyelinated neurons by noxious stimuli causes the simultaneous release of neuropeptides such as SP, neurokinin A (NKA), neurokinin B (NKB), and calcitonin gene-related peptide (CGRP) [69]. Their release invokes neurogenic inflammation, a neurally elicited response with the typical features of an inflammatory response involving vasodilation and increased vascular permeability [70]. CGRP is chiefly responsible for promoting vasodilation, while SP primarily induces plasma extravasation, although it also produces a brief period of vasodilation [71]. Indeed, although NKA, NKB, and SP act synergistically [72], increases in capillary permeability are principally mediated by SP [73, 74].

SP is an 11-amino acid peptide that is a member of the tachykinin family which includes NKA and NKB [75]; both NKA and SP derived from the preprotachykinin-A (PPT-A) gene by alternative splicing [76]. SP is widely distributed throughout the CNS, peripheral nervous system (PNS), and enteric nervous systems. In the CNS, it is present in dorsal root ganglion (primary sensory) neurons [77] of the spinal cord and many regions of the brain including the hippocampus, cortex, basal ganglia, hypothalamus, amygdala, and caudate nucleus, being more abundant in the gray matter compared to the white matter [78]. The biological effects of SP are mediated by the tachykinin NK receptors, with SP preferentially binding to the NK1 receptor, although it has some affinity for the NK2 and NK3 receptors. NK1 receptors are expressed on endothelial cells, astrocytes, microglia, and various types of circulating and inflammation-activated immune cells [79]. Transduction of the SP signal through the NK1 receptor occurs via G protein signaling and the secondary messenger cAMP, ultimately leading to the regulation of ion channels, enzyme activity, and alterations in gene expression [80]. There are two versions of the NK1 receptor: the full-length version and a truncated form which lacks the 96 residues at the C terminus [81]. This truncated form has a diminished binding affinity for SP [79], and its activation produces a much diminished inflammatory response when compared to the full-length receptor [82]. Higher levels of expression of the shorter isoform are found in peripheral tissues, while in the brain, the longer isoform is expressed at much higher concentrations than the truncated version [83]. This suggests that for pathology within the CNS, the full-length version of the NK1 receptor is the most critical.

### Role of SP following TBI

Extensive research has shown that levels of SP rise acutely following TBI in both pre-clinical animal models and in human tissue. Virtually, all blood vessels of the body are surrounded by sensory nerve fibers that contain SP [84]. Cerebral arteries, in particular, appear to receive a dense supply of these nerve fibers, and our studies in TBI have demonstrated that perivascular SP immunoreactivity increases in pre-clinical models, irrespective of injury model [13, 85, 86], and also in humans [87]. It appears that SP is released early following TBI, with increases noted in the plasma at 30 min following TBI in rodents [13]. Furthermore, this release of SP appears to depend on the magnitude of the insult, with a graded increase in SP immunoreactivity seen with increasing severity of injury [85, 88]. Indeed, SP appears to be a key injury marker, as levels in the plasma over the first 4 h following injury are significantly correlated with early mortality in clinical populations, with non-surviving TBI patients showing significantly higher levels than survivors [88].

Moreover, it has been shown that attenuating SP activity following TBI is beneficial to outcome [89]. The first demonstration of neurogenic inflammation in TBI showed that depletion of sensory neuropeptides by pre-treatment with capsaicin results in the attenuation of post-traumatic BBB permeability, edema formation, and improved functional outcome [11]. Later studies, specifically targeted SP by administering an NK1 antagonist showed beneficial effects in both male [13] and female rats [90], with a significant attenuation of post-traumatic BBB permeability and a resultant significant reduction in edema formation with improvement in motor and cognitive outcome.

### What promotes the release of SP following TBI?

It appears likely that the initial release of SP from sensory neurons following TBI may be mediated by mechanical activation of members of the transient receptor potential (TRP) family, predominantly TRPV1 and TRPA1 [91]. Like all TRP receptors, TRPV1 and TRPA1 are comprised of six-transmembrane proteins that assemble as tetramers to form cation-permeable pores [92, 93]. Their activation allows the influx of cations, primarily sodium and calcium, triggering the release of neuropeptides [94, 95]. TRPV1 appears to be co-expressed in most if not all of TRPA1 expressing dorsal root ganglion neurons [96, 97], with co-expression of both these receptors with neuropeptides including SP [98]. Indeed, suppression of the TRPV1 receptor, with the antagonist capsazepine, has been shown to significantly reduce SP levels in a number of inflammatory models, including a model of sepsis [99], alcohol-induced gastric injury [100], and formalin-induced asthma [101], with the latter study showing a similar reduction in SP with administration of the TRPA1 antagonist, HC-030031 [101]. Furthermore, activation of both TRPV1 and TRPA1 [102–104] has been linked to increased vascular permeability, a key downstream effect of SP release. TRPV1 immunoreactivity is prominent in astrocytes and pericytes, which are closely associated with the vasculature, as well as neurons [105], with the administration of capsazepine able to reduce BBB disruption following an ischemia-reperfusion injury [106].

TRPV1 and TRPA1 channels are considered as polymodal receptors that are activated by a wide range of stimuli. For TRPV1, this includes capsaicin (the active ingredient in chilies) [107], heat (43–52 °C) [108], protons [107], bradykinin [109], prostaglandins [110], and arachidonic acid metabolites amongst others [111]. For TRPA1, agonists include exogenous noxious agents such as components of wasabi and cinnamon [98], oxidized lipids [112], protons [113], and potentially cold (<17 °C) [113, 114]. Notably, both TRPV1 and TRPA1 are also putative mechanoreceptors. Early reports identified that the long ankyrin repeat region within the N-terminal domain of TRPA1, which assists in anchoring the receptor to the plasma membrane, could form a spring-like structure to sense mechanical forces [115, 116]. Indeed, TRPA1 knockout mice were found to be less sensitive to low-intensity mechanical stimuli compared to wild-type mice, and responses to high-intensity mechanical stimulation were notably impaired [113]. Furthermore, inhibition of TRPA1 via either gene knockout or treatment with the antagonist HC-03001 reduced mechanically induced action firing in dermal C fibers [117, 118], wide dynamic range, and nociceptive-specific neurons within the spinal cord [119] and mechanosensitive visceral afferents in the colon [120, 121]. Studies have also suggested that TRPV1 detects mechanical stimuli in a variety of tissues including the urothelium cells of the bladder [122], colonic primary afferent neurons [123], renal pelvis [124], and retinal ganglion cells [125, 126]. The threshold for the stimulation of both TRPV1 and TRPA1 appears to be lowered when there is pre-existing inflammation. Indeed, blockade of TRPA1 was only effective in reducing low-intensity mechanical stimulation of spinal neurons in animals with inflammatory arthritis [119], and application of a TRPV1 antagonist reduced Aδ-fiber unit responses only in inflamed skin [127].

In regard to TBI, a report by Zacest et al. showed that TBI mechanical stimulation of sensory nerves, presumably via TRPV1 and TRPA1, facilitates the perivascular release of SP, with SP co-localizing with amyloid precursor protein positive (injured) neurons that supply the blood vessels [87]. Apart from the initial external mechanical insult to the brain, another acute mechanical stimulus could be related to a brief but highly significant spike in blood pressure (BP) seen following injury. In a sheep model of TBI, BP was reported to rise markedly immediately after impact, peaking at a MAP of 176 mmHg before gradually returning to baseline over a 10-min period [128]. Similar findings have been shown in other models of experimental TBI [129–131]. TRPV1 receptors have indeed been shown to respond to increased intraluminal pressure [132, 133], with Scotland et al. showing that the normal myogenic response to elevation of increased transmural pressure in mesenteric small arteries in vitro was suppressed with the application of the TRPV1 antagonist capsazepine. As such release of neuropeptides including SP may be one of the earliest responses to TBI, highlighting its integral role in facilitating the inflammatory response.

## How does neurogenic inflammation potentiate classical inflammation?

### Direct interaction between SP and the classical inflammatory response

**Fig 1:**
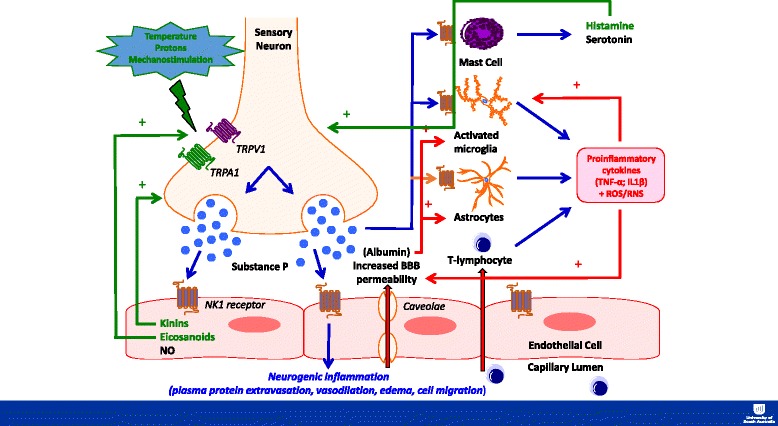
Interaction between the neurogenic and classical inflammatory response following TBI

SP induces and augments many aspects of the classical inflammatory response (Fig 1), including leukocyte activation, endothelial cell adhesion molecule expression, and the production of inflammatory mediators such as histamine, nitric oxide, cytokines (such as IL-6), and kinins [14, 15]. SP is a potent mast cell activator [134]. Mast cells are found within the brain on the adluminal side of the BBB and in the leptomeninges [135], with their degranulation found to potentiate excitotoxicity [136] and augment and prolong numerous vasoactive, neuroactive, and immunoactive cellular and molecular responses to injury [137, 138].

SP also directly activates microglia and astrocytes. Injury induces the expression of NK1 receptors on astrocytes, and their activation is thought to contribute to the transformation to reactive astrocytes, with the resultant production of inflammatory mediators such as cytokines, prostaglandins, and thromboxane derivatives [139–141]. Similarly SP can promote microglial activation, initiating signaling via the NFκB pathway, which leads to the production of pro-inflammatory cytokines [142], with microglia producing IL-1 in response to SP [143]. Moreover, following TBI, NK1 antagonists have been shown to significantly reduce the production of the pro-inflammatory cytokine IL-6, as well as to decrease microglial proliferation [144].

### Role of SP in altering BBB permeability

Apart from directly augmenting the local classical inflammatory response, SP-related BBB disruption may also play a role in perpetuating the inflammatory response. The BBB is a highly selective barrier formed by a layer of endothelial cells joined together by tight junctions including proteins such as claudins, occludin, junctional adhesion molecules (JAMs), and zonula occluden (ZO) proteins, which are supported by the end-feet of astrocytes that act to support and enhance the tight junctions [145]. Tight junctions are present at the apical end of the interendothelial junction and closely rely on the integrity of the corresponding cadherin junction located in the basolateral region. Ultrastructural studies of cerebral endothelial cells indicate the circumferential arrangement of the TJs which provide the barrier formation required to actively restrict the movement of small hydrophilic molecules such as sodium and particular permeability tracers between cells [146]. Given the restrictions on paracellular transport, cerebral endothelial cells have tightly controlled mechanisms that enable the bidirectional transcellular passage of essential molecules and the efflux of potentially neurotoxic substances and waste products [147]. Smaller molecules can be ferried across via carrier-mediated transport or ion pumps while macromolecules employ transcytosis, which can be either receptor-mediated transport (RMT) or adsorptive-mediated transport (AMT), where positively charged macromolecules are nonspecifically transported across the cerebral endothelium [148]. Although the cerebral endothelium has relatively few pinocytotic vesicles compared to peripheral endothelial cells [149], they can still transport macromolecules via one of three vesicles: clathrin-coated pits, the most numerous and through which most of the RMT occurs, the smaller and less numerous caveolae, capable of both AMT and RMT, and large macropinocytic vesciles with nonspecific cargo [150].

Following TBI, the BBB is disrupted and this encompasses not just potential disruption to tight junctions but also increased transcytosis and altered transport properties [151–153]. This increase in the transcellular permeability of the BBB permits the extravasation of proteins and solutes from the cerebral vasculature into the extracellular space within the brain, promoting edema formation, perpetuating the inflammatory response, and causing further neuronal injury. Importantly, it appears that this transcytotic activity is the initial alteration to the BBB following TBI, which is then followed by later loss of tight junctions and movement via the paracellular pathway [151, 152]. An early study by Povlishock (1978) found an increase in BBB permeability as early as 3 min following a mild central fluid percussion injury, despite the apparent integrity of the endothelial tight junctions [154]. Notably, endothelial vesicles assume a modified appearance and initiate the formation of extensive networks for intracellular transport after injury, particularly in the setting of inflammation within the CNS [155]. Indeed, ultrastructural studies have shown that within minutes of brain injury, endothelial caveolae are significantly increased in vessel segments showing loss of BBB integrity [152, 155], with evidence for increased vesicular formation in the BBB of TBI patients [156, 157]. In a rat cortical cold injury model that produces a pure vasogenic edema, an increase in expression of caveolin-1, the key protein found in caveolae, occurred prior to the loss of tight junctions, represented by a reduction in the expression of occludin and claudin-5 [152]. Notably, caveolin-1 expression was seen in blood vessels which exhibited increased permeability to proteins, indicating that this may be the initial mechanism by which the BBB is disrupted [152]. These findings were replicated in a controlled cortical impact model of TBI, where caveolin-1 was increased at 1 day following injury, prior to a decrease in claudin-5 which occurred at day 3 following injury [151].

SP is known to promote increased permeability of the BBB leading to increased extravasation of vascular protein into the brain extracellular space. Indeed, injured animals show a strong co-localization of SP immunofluorescence with a marker of vascular permeability, Evan’s blue, within the cortical vasculature at 5 h post-TBI [13]. Administration of an NK1 antagonist also leads to a dose-dependent decrease in permeability of the BBB following TBI, with an accompanying reduction in cerebral edema [13]. The exact mechanisms by which SP promotes an increase in BBB permeability are less clear. There are previous reports that application of SP can decrease the expression of ZO-1 and claudin-5 in cerebral capillary endothelial cells [158], but immediately following TBI tight junctions are intact. Alternatively, SP may initially act to increase transcytosis via activation of transport via caveolae.

Caveolae have been shown to mediate the transport of molecules such as albumin, transferrin, insulin, low-density lipoproteins, cytokines, and chemokines through the specific localization of corresponding receptors within the caveolae coats [159, 160]. Indeed, caveolin-1 knockout mice were unable to transcytose albumin as evaluated via gold-labeled immunostaining [161, 162], supporting a key role for caveolae in the early BBB changes seen following TBI, and consistent with the increases seen in expression of caveolin-1 in the immediate phase following injury [151–153]. Caveolae constitute an entire membrane system responsible for the formation of unique endo- and exocytotic compartments [163]. During transcytosis, caveolae “pinch off” from the plasma membrane to form vesicular carriers that rapidly and efficiently shuttle to the opposite membrane of the endothelial cells, fuse and release their contents via exocytosis [164]. In addition to the structural caveolin proteins, a number of signaling molecules, including specific receptors, are known to localize to the caveolae pit [160, 165]. Receptor tyrosine kinase, G-protein-coupled receptors, transforming growth factor-beta (TGF-β) type 1 and 2, certain steroid receptors and enzymes have a confirmed presence within endothelial caveolae and may play a role in facilitating macromolecular transcytosis [166–168]. Of particular note, the NK1 receptor, to which SP preferentially binds, has been reported to localize within the endothelial caveolae and can be manipulated to upregulate or relocate upon stimulation, indicating that its presence and function is dynamic and subject to the environment [169, 170]. Indeed, stimulation of the NK1 receptor within caveolae by SP causes PKCα to relocate to caveolae [170], with this process previously shown to regulate the internalization of caveolae [171], the first step in transcytosis. Although not directly studied for the NK1 receptor, in vitro studies have demonstrated increased receptor-mediated transendothelial transport of target molecules in the setting of inflammation [172]. This suggests that the activation of a caveolae-housed receptor may also internalize additional proteins in their cargo, such as albumin, facilitating their entry into the brain.

Within the CNS, albumin is able to activate microglia and astrocytes leading to the release of pro-inflammatory cytokines and chemokines (e.g., IL-1β, TNFα, MCP-1, CXC3L1), glutamate, and free radicals promoting neuronal injury and amplifying the classical inflammatory response [173–178]. Although the majority of these studies apply albumin to isolated glial cultures [174–177], Chacheaux et al. were able to demonstrate that direct application of a solution containing bovine serum albumin to the rat cortex produced similar changes in gene expression when compared to application of an agent (deoxcycholic acid) that directly opens the BBB [179]. Microarray analysis showed a comparable gene expression profile to either treatment, with an early and persistent upregulation of genes associated with immune response activation, including NF-κB pathway-related genes, cytokines, and chemokines (IL-6, CCL2, CCL7) [179]. Indeed, recent evidence suggests that following activation by albumin, microglia are neurotoxic, with media taken from microglial cultures exposed to albumin promoting the upregulation of caspase 3 and 7 in cerebellar granule neuronal cultures with an accompanying increase in cell death [174]. Furthermore, when exposure to albumin was combined with TLR4 activation via lipopolysaccharide (LPS), a synergistic increase in the release of pro-inflammatory cytokines was noted from cultured microglia [174]. As the neuronal injury associated with TBI leads to the production of DAMPs [180] that act as TLR4 agonists, influx of albumin through a disrupted BBB would lead to more potent activation of microglia. This provides another key intersection between the classical inflammatory response, in TLR4 activation by DAMPs, and the neurogenic inflammatory response, with SP release promoting albumin influx via caveolae promoting further activation of resident immune cells.

The effects of SP on the BBB relates not only to increases in permeability but also on promoting immune cell trafficking into the CNS. Activation of SP’s preferred receptor, NK1, has been shown to enhance leukocyte migration by exerting a chemotactic effect on monocytes and neutrophils [181–183], increasing the expression of adhesion molecules on endothelial cells [184–187] and augmenting local chemokine production [188]. Indeed, application of SP to cerebral endothelial cultures led to a dose-dependent increase in ICAM-1 expression [184], with this associated with an increase in T lymphocyte adherence, suggesting facilitation of immune cell movement across the BBB [187]. Thus, the release of SP following TBI may facilitate the influx of peripheral immune cells like T cells, macrophages, and neutrophils into the CNS, which then further augments the local neuroinflammatory response via production of pro-inflammatory cytokines, reactive oxygen species, and metalloproteinases.

## Exacerbation of neurogenic inflammation by classical inflammation

Although this discussion has focused on how neurogenic inflammation can facilitate the classical inflammatory response, this is a two-way interaction with classical inflammation further enhancing the release of neuropeptides (Fig 1). As discussed previously, TPRV1 and TRPA1 are responsive to a number of stimuli including the classical inflammatory mediators bradykinin [109] and prostaglandins [110], which are produced as part of the inflammatory response following TBI [189, 190]; their production would potentiate further SP release. For example, the kallikrein-kinin system is believed to be one of the first inflammatory pathways activated after tissue damage [191]. Bradykinin, a nonpeptide found in blood and tissue, is cleaved from its pro-form kininogen by the protease kallikrein [192], with levels found to peak within the brain at 2 h following a focal brain injury [193]. TBI is also known to increase the activity of cyclooxygenase (COX) enzymes, principally Cox-2 found in microglia and endothelial cells, increasing the synthesis of prostaglandins such as PGE_2_ and PGF_2α_. Indeed, levels of PGE_2_ have been shown to be elevated within 5 min following TBI [194]. In addition to these direct activators or TRP channels, cytokines like IL-1, IL-6, and TNFα may sensitize sensory neurons, lowering the threshold for release of neuropeptides [195, 196].

## Conclusion

Following TBI activation of a classical inflammatory response only represents part of the neuroinflammatory response. The mechanical and shear stress associated with TBI also activates TRP receptors leading to the release of neuropeptides, including SP, instigating a neurogenic inflammatory response. SP both directly and indirectly, through alterations in BBB permeability with influx of plasma proteins, augments the classical inflammatory response, with both classical and neurogenic inflammation providing positive feedback to the other to amplify and propagate inflammation with the release of pro-inflammatory mediators, oxidative metabolites, and metalloproteinases amongst others, which cause further neuronal damage. As such, modulation of neuroinflammation following TBI may require addressing both inflammatory pathways with the aim to prevent the deleterious effects of the response, while facilitating repair.

## Abbreviations

AMT: Absorptive-mediated transcytosis
BBB: Blood-brain barrier
CNS: Central nervous system
EPO: Erythropoietin
RMT: Receptor-mediated transcytosis
SP: Substance P
TLR: Toll-like receptor
TRP channels: Transient receptor potential cation channels
TBI: Traumatic brain injury
